# Room Temperature Negative Differential Resistance with High Peak Current in MoS_2_/WSe_2_ Heterostructures

**DOI:** 10.1021/acs.nanolett.3c04607

**Published:** 2024-02-16

**Authors:** Jung Ho Kim, Soumya Sarkar, Yan Wang, Takashi Taniguchi, Kenji Watanabe, Manish Chhowalla

**Affiliations:** † Department of Materials Science and Metallurgy, University of Cambridge, 27 Charles Babbage Road, Cambridge CB3 0FS, United Kingdom; ‡ Research Center for Materials Nanoarchitectonics, National Institute for Materials Science, 1-1 Namiki, Tsukuba, Ibaraki 305-0044, Japan; § Research Center for Electronic and Optical Materials, National Institute for Materials Science, 1-1 Namiki, Tsukuba, Ibaraki 305-0044, Japan

**Keywords:** 2D materials, negative differential resistance, tunnel transistor, MoS_2_/WSe_2_ heterostructure, *h*-BN tunnel barrier

## Abstract

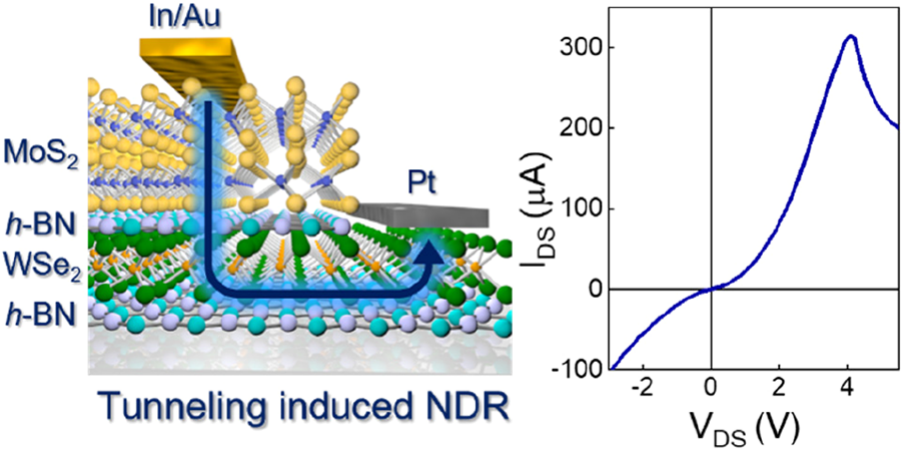

Two-dimensional transition metal dichalcogenide (2D TMD) semiconductors allow facile integration of p- and n-type materials without a lattice mismatch. Here, we demonstrate gate-tunable n- and p-type junctions based on vertical heterostructures of MoS_2_ and WSe_2_ using van der Waals (vdW) contacts. The p–n junction shows negative differential resistance (NDR) due to Fowler–Nordheim (F–N) tunneling through the triangular barrier formed by applying a global back-gate bias (*V*_GS_). We also show that the integration of hexagonal boron nitride (*h*-BN) as an insulating tunnel barrier between MoS_2_ and WSe_2_ leads to the formation of sharp band edges and unintentional inelastic tunnelling current. The devices based on vdW contacts, global *V*_GS_, and *h*-BN tunnel barriers exhibit NDR with a peak current (*I*_peak_) of 315 μA, suggesting that the approach may be useful for applications.

Devices with negative differential resistance (NDR) exhibit multiple threshold voltages, making them attractive for multivalue logic systems^1,2^ and radio frequency oscillators.^3^ NDR devices are based on the transition of carrier transport from quantum mechanical tunnelling to thermionic emission by sweeping the applied voltage.^4^ To use NDR for functional devices, the output characteristics of the transistor in the NDR regime should have a high peak current (*I*_peak_) and a high peak-to-valley current ratio (PVCR). The importance of high *I*_peak_ and high PVCR lies in their role in enabling efficient signal amplification, reliable switching behavior, and optimization of device performance for various high-frequency applications.^5,6^

Two-dimensional transition metal dichalcogenide (2D TMD) semiconductors are ideally suited for realizing NDR because they can be easily assembled, and sharp interfaces can be formed without lattice mismatch.^7^ A variety of NDR devices based on heterostructures of 2D semiconductors have been reported. In particular, heterostructures using SnSe_2_^8−10^ or black phosphorus (BP)^11,12^ have been studied because both are highly doped degenerate 2D semiconductors—SnSe_2_ being n-type and BP being p-type. Despite the ease of forming type III band alignment due to the degeneracy, the low ambient stability and consequential surface oxidation make it difficult to achieve clean interfaces.^8^ Conversely, widely used 2D TMDs such as MoS_2_ and WSe_2_ can provide improved ambient stability. Thus, several reports have explored tunnel devices using MoS_2_ and WSe_2_ heterostructures. Roy et al. have reported a dual-gate MoS_2_/WSe_2_ heterojunction, which shows NDR behavior at low temperatures.^13^ Here, the dual-gate structure plays a key role in electrostatically doping the two materials to split the band alignment. Additionally, Nourbakhsh et al. have shown room-temperature (RT) NDR with MoS_2_/WSe_2_ heterostructure with back-gate bias (*V*_GS_) modulation.^1^ Through calculated band diagrams, the authors determined the optimal thicknesses of the two materials that would allow tunneling in the transverse direction. However, the low *I*_peak_ of a few hundred pA should be improved for practical application.

In this work, we demonstrate RT gate-tunable MoS_2_ and WSe_2_ heterostructures. We use In/Au van der Waals (vdW) contacts for n-type transport in MoS_2_^14^ and Pt vdW contacts for p-type transport in WSe_2_,^15^ which help in boosting electron and hole injection, respectively. We compare the performance of the MoS_2_/WSe_2_ junctions when they are directly in contact with each other and when a thin hexagonal boron nitride (*h*-BN) tunnel barrier is inserted between them. We observe that the devices with a *h*-BN tunnel barrier and vdW contacts exhibit an *I*_peak_ of 315 μA, which is among the highest values reported at RT.

For the NDR, forming an effective p–n junction is necessary. We applied three strategies to form an effective p–n junction. First, for all devices, we have used WSe_2_ consisting of two or three layers and MoS_2_ of >10 layers, fabricated on *h*-BN on SiO_2_ (90 nm)/Si substrates (device fabrication is described in detail in the Supporting Information, Experimental Methods). We selected the layer thicknesses based on an earlier study, which calculated the optimal thicknesses for NDR.^1^ The use of *h*-BN as a substrate helps reduce hysteresis due to substrate traps,^16^ interface phonon scattering,^17^ and most importantly electron doping from SiO_2_,^15^ essential for achieving p-type (WSe_2_) and improving device performance (Figure S1). Finally, we used In (8 nm)/Au (80 nm) and Pt (20 nm capped with 60 nm of Au) vdW contacts for the MoS_2_ and WSe_2_ FETs, respectively. The optical microscope (OM) image of MoS_2_ and WSe_2_ FETs with vdW contacts is shown in Figure 1a. Our prior investigation revealed that vdW contacts, specifically In/Au on MoS_2_ and Pt on WSe_2_, exhibit distinct n- and p-type transport behavior, originating from clean metal/semiconductor interfaces that prevent Fermi-level pinning (FLP).^14,15^

**Figure 1:**
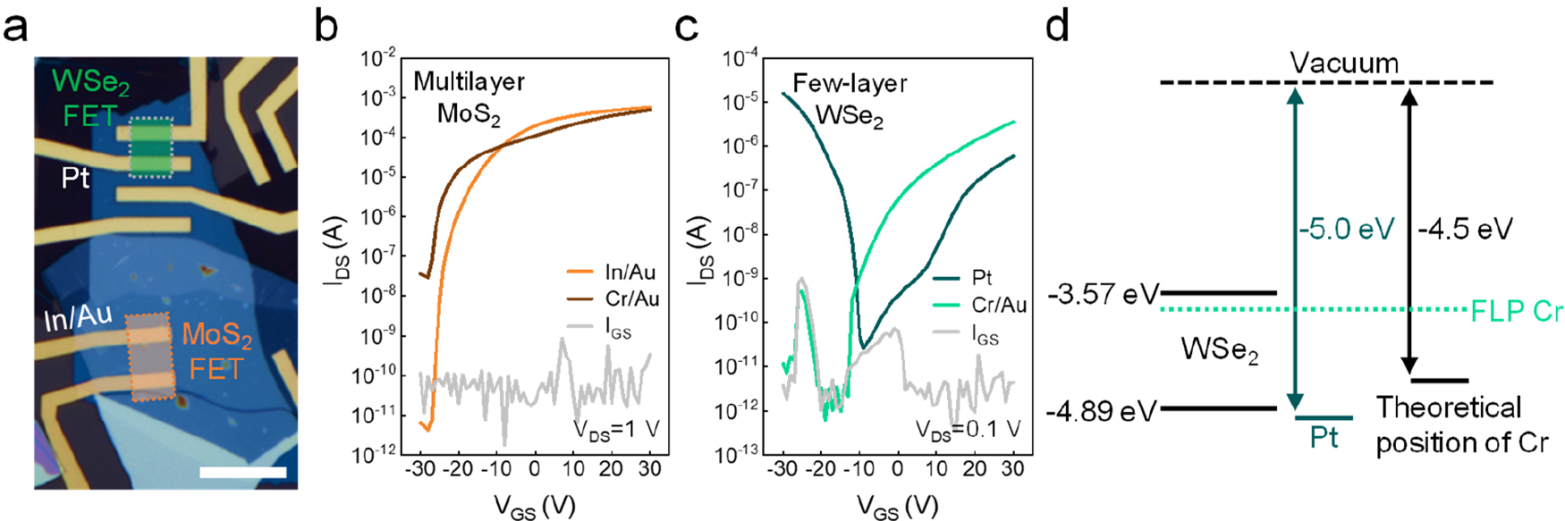
FET comparison of vdW and Cr/Au contacts. (a) OM image of MoS_2_ and WSe_2_ FETs. The scale bar is 10 μm. (b,c) *V*_GS_-dependent transfer curves of (b) multilayer MoS_2_ and (c) few-layer WSe_2_. Gray-colored curves represent gate-leakage current (*I*_GS_). (d) Energy band alignment of WSe_2_ and the metal contacts. FLP of Cr contacts induce n-type transport of WSe_2_.

We compared the characteristics of FETs with vdW contacts with non-vdW contacts based on Cr (5 nm)/Au (80 nm), commonly used for 2D TMD FETs (Figure 1b and c). Both In/Au and Cr/Au contacts show n-type behavior,^18^ while In/Au contacts show an improved on/off ratio. In contrast, Cr/Au contacts on few-layer WSe_2_ exhibit n-type transport while the vdW Pt contacts show p-type-dominant behavior.^19^ This result is consistent with previous studies showing that vdW contacts are effective in achieving p-type transport in WSe_2_.^15,20^ In addition, temperature-dependent transport measurements showing stable transport behavior over a large temperature range are shown in Figure S2.

The p-type behavior with Pt vdW contacts can be understood by using the energy band diagram in Figure 1d. According to the band alignment, Cr/Au contact is expected to show ambipolar transport closer to p-type.^21,22^ However, for non-vdW contacts, defects are formed at the metal/semiconductor interface leading to FLP close to the conduction band (CB) of WSe_2_,^23^ which leads to n-type behavior of Cr/Au-WSe_2_ FETs.^24^ In contrast, Pt deposition forms a vdW gap between the metal and WSe_2_, which follows the Pt work function. This leads to an effective hole injection for p-type WSe_2_.

Figure 2 describes the role of vdW contacts in heterostructure performance. We prepared two different sets of devices with identical configurations: a stack of multilayer MoS_2_ flakes on top of few-layer WSe_2_ on the *h*-BN bottom layer. In the first set of devices, we deposited In and Au on MoS_2_ and Pt on WSe_2_. Figure 2a displays an OM image of a device along with a device schematic at the bottom. For comparison, we fabricated another set of devices with Cr/Au contacts (see Figure S3 for an OM image of such a device). We applied voltage (drain-source voltage, *V*_DS_) to WSe_2_ (drain) while MoS_2_ (source) was grounded. The current–voltage (*I*–*V*) curves of these two types of devices, measured at RT, are plotted in Figure 2b (the *V*_GS_-dependent *I*–*V* curves are presented in Figure S4). The devices with vdW contacts exhibit a clear NDR, whereas it is absent in devices with Cr/Au contacts. The observation of NDR in the devices with vdW contacts is evidence of tunneling (the *I*_DS_–*V*_GS_ curve is depicted in Figure S5).

**Figure 2:**
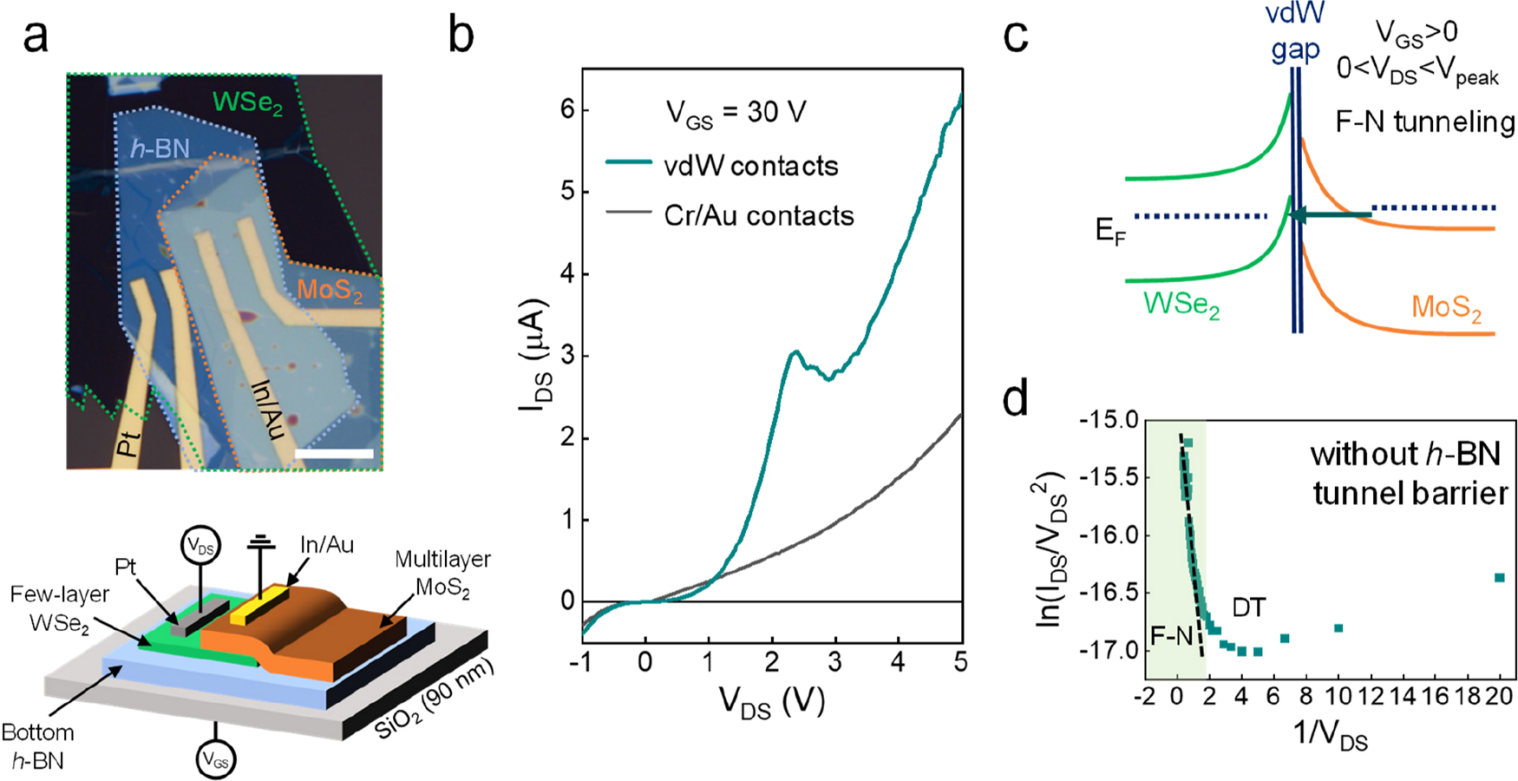
A comparison of NDR from FETs with vdW and Cr/Au contacts. (a, top) OM image of MoS_2_/WSe_2_ heterostructure devices. In/Au and Pt electrodes were used as electrical contacts on MoS_2_ and WSe_2_, respectively. The scale bar is 10 μm. (bottom) A device schematic drawing of the device. (b) *I*–*V* curves of the vdW (green) and Cr/Au contacts (black). (c) Energy band diagram of the MoS_2_/WSe_2_ heterojunction with a vdW gap and F–N tunnelling. The drawing is based on *V*_GS_ > 0, 0 < *V*_DS_ < *V*_peak_ conditions. (d) ln(*I*_DS_/*V*_DS_^2^) versus 1/*V*_DS_ plot. Linear region (black dotted line) refers to the F–N tunnelling.

To observe NDR in the forward bias region (*V*_DS_ > 0) of a p–n junction, a type III junction, with a broken band alignment at *V*_DS_ = 0 V is needed. Type III junctions can form when both p- and n-type semiconductors are highly doped and are in a degenerate state. However, the materials that we used (MoS_2_ and WSe_2_) are not in a degenerate state. Moreover, unlike a dual gate structure,^13^ global *V*_GS_ makes it difficult to control the carrier density of each material separately. Notably, in our heterostructures, NDR shows a more distinct trend at *V*_GS_ > 0, which is also observed in previous works.^12,25^ We attribute this to steep band bending of a few layers of WSe_2_ with better *V*_GS_ coupling due to its proximity to the gate and slight bending of multilayer MoS_2_ due to screening from WSe_2_ (Figure 2c). At 0 < *V*_DS_ < 2.35 V (peak voltage, *V*_peak_), the electrons from the MoS_2_ tunnel through the thin triangular barrier and reach WSe_2_. With increasing forward bias, the overlap between the MoS_2_ CB and the WSe_2_ VB closes the thin triangular tunnel path, and therefore the tunneling current begins to decrease, thereby showing NDR. Conversely, the case differs when Cr/Au contacts are employed. Because the Fermi-level is pinned, WSe_2_ band bending cannot occur effectively under *V*_GS_ variation. Hence, the MoS_2_ electrons will face the midband gap state of WSe_2_ due to the forming of a small triangular barrier. The tunnel transport mechanism is depicted in Figure 2d to validate the tunnel transport mechanism. The *I*–*V* curve in the region of 0 < *V*_DS_ < *V*_peak_ can be modeled using the Simmons approximation.^26^ The Fowler–Nordheim (F–N) tunnelling can be expressed as
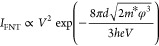
Here, *d* represents the thickness of the tunnel barrier, m* denotes the effective electron mass, φ corresponds to the tunnelling barrier height, and *h* is the Planck constant. Plotting ln(*I*/*V*^2^) versus 1/*V* should reveal a linear regime with a negative slope (black dotted line) for F–N tunnelling.^27,28^ From the slope of the curve, we can derive the F–N tunneling barrier height (φ) of 0.11 eV.

We further validate the occurrence of F–N tunneling in our devices through temperature-dependent transport measurements. In Figure S6, we present the temperature-dependent *I*–*V* curves from a device showing NDR with In/Au and Pt contacts. Here, we can observe that the steep increase of tunnelling current in the reverse bias region (*V*_DS_ < 0 V) is insensitive to changes in temperature. Furthermore, the F–N tunnelling current, which is the increase in current in the forward bias region preceding the NDR effect (0 < *V*_DS_ < *V*_peak_), also shows a negligible temperature dependence. However, beyond the NDR effect, the current flow is governed by thermionic emission and is highly sensitive to changes in temperature (Figure S6, inset). The results in Figures 2b,d and S6 suggest that the vdW gap between MoS_2_/WSe_2_ alongside the contribution of F–N tunneling is critical.

Next, we integrated 10 layers (3.4 nm) of *h*-BN between MoS_2_ and WSe_2_. The insertion of *h*-BN as a tunnel barrier serves as an insulating spacer that enables precise band alignment.^17,29,30^ The insulating nature of *h*-BN results in sharper band edges for both MoS_2_ and WSe_2_. Consequently, the energy levels of the VB and CB experience a sharp transition at the MoS_2_/*h*-BN and WSe_2_/*h*-BN interfaces, resulting in a well-defined tunnel barrier. However, at the same time, it requires extra energy for the electrons to tunnel through the barrier. Also, due to additional stacking steps during fabrication and natural defects in the *h*-BN crystal, unintentional trap sites may be involved.^31^ The difference in the transport behavior between two devices with and without the *h*-BN tunnel barrier at RT is shown in Figure 3a. Both devices show clear NDR behavior. However, the device with the *h*-BN tunnel barrier (left *y* axis, orange) displays a substantial increase (2 orders of magnitude) in *I*_peak_ compared to the device without the tunnel barrier (right *y* axis, green). Additionally, we observe an increase in the *V*_DS_, from 2.35 to 4.1 V, required to reach the *I*_peak_. We attribute this to the presence of an additional tunnel barrier (*h*-BN), which necessitates a higher voltage for carriers to reach the opposite side (WSe_2_) of the energy band.

**Figure 3:**
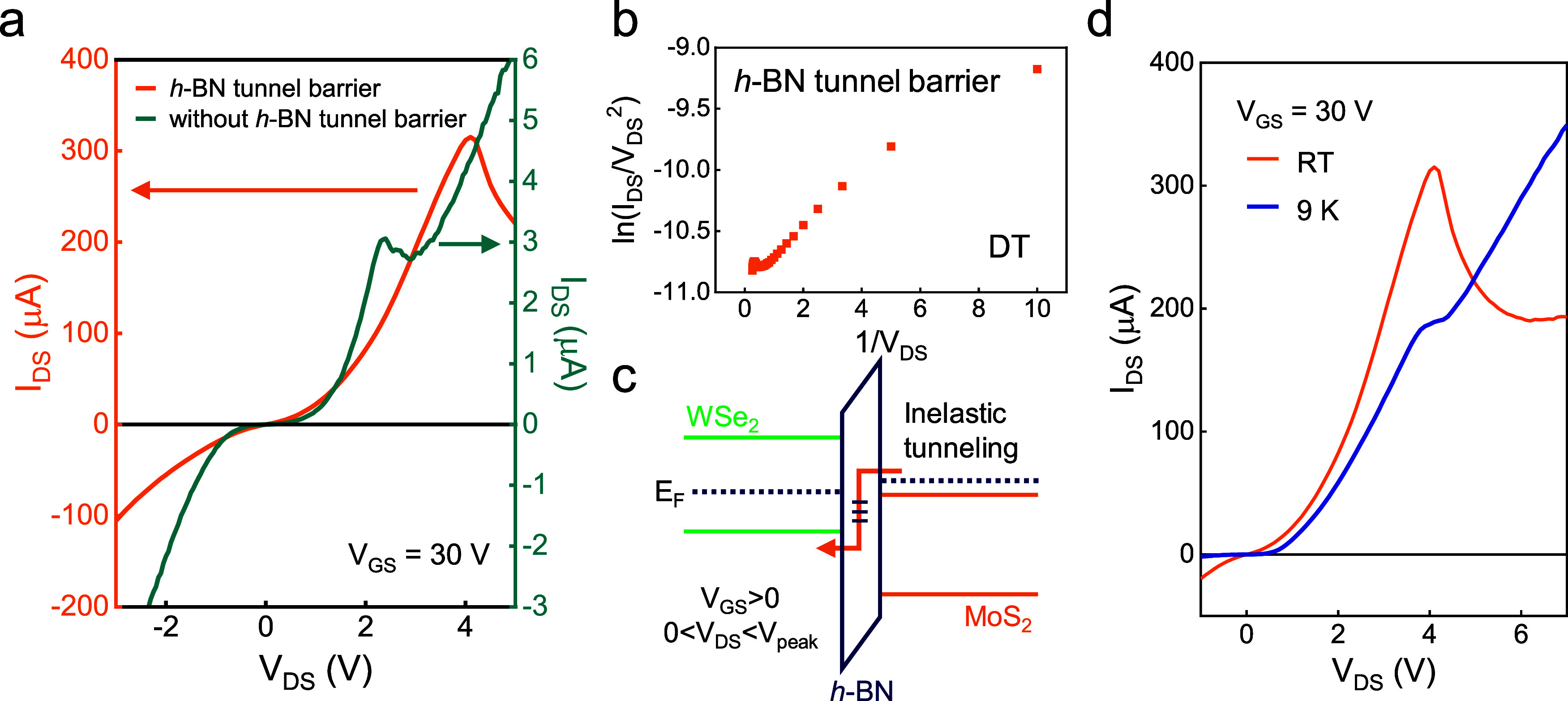
The role of the *h*-BN tunnel barrier. (a) *I*–*V* curves with *h*-BN tunnel barrier (orange) and without tunnel barrier (green). (b) ln(*I*_DS_/*V*_DS_^2^) versus 1/*V*_DS_ plot depicting DT for the device with an *h*-BN tunnel barrier. (c) Energy band diagram of MoS_2_/WSe_2_ heterojunction with *h*-BN tunnel barrier. The drawing is based on *V*_GS_ > 0 and 0 < *V*_DS_ < *V*_peak_ conditions. (d) NDR behavior of the device with *h*-BN tunnel barrier at 9 K and RT.

To explore the mechanism for higher *I*_peak_, the *I*–*V* curve in the forward bias region (0 < *V*_DS_ < *V*_peak_) is studied. Unlike the F–N tunnelling observed in Figure 2d, Figure 3b follows a logarithmic slope, meaning the device incorporating the *h*-BN tunnel barrier operates via direct tunnelling (DT).^32^ The DT current can be expressed as
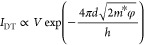


The possible energy band alignment due to DT transport through the *h*-BN tunnel barrier is shown in Figure 3c. Although precise band alignment is difficult to extract and varies from study to study,^1,13,27^ we can imagine the presence of inelastic tunnelling such as phonon-assisted, trap-assisted, or Shockley–Read–Hall tunnelling.^13,33,34^ To obtain more insight, we plotted the NDR behavior of the *h*-BN tunnel barrier device at 9 K and RT for comparison (Figure 3d). It shows a distinct increase in the current at RT, and such a temperature-dependent enhancement is absent in devices without an *h*-BN tunnel barrier (Figure S6). This leads to a higher *I*_peak_ and a larger peak-to-valley current ratio (PVCR) at higher temperatures due to thermally enhanced inelastic tunnelling. Thus, we believe that combined effects including the sharp band edges as well as inelastic tunneling through the *h*-BN barrier are important for the enhanced *I*_peak_.

The properties of devices with the *h*-BN tunnel barrier were further examined by varying *V*_GS_. Figure 4a shows an OM image of a typical device. The three-layer thickness of WSe_2_ enables effective gate tunability of the Fermi level, while the effect of the *V*_GS_ on the relatively thick (10 layers) MoS_2_ is small. The thicknesses of the layers measured by atomic force microscopy (AFM) topography are presented in Figure S7. Electrical bias was applied to the Pt contact connected to WSe_2_, while the current was collected from the In/Au contact on MoS_2_. The gate bias was applied by global *V*_GS_ (SiO_2_/Si). The *V*_GS_ dependence of the NDR at RT is plotted in Figure 4b. With increasing *V*_GS_, the NDR becomes more pronounced, resulting in higher current levels.^12,25^ This behavior can be attributed to the accumulation of electrons in MoS_2_ as *V*_GS_ > 0, providing a large number of carriers available for tunnelling when a forward bias is applied.

**Figure 4:**
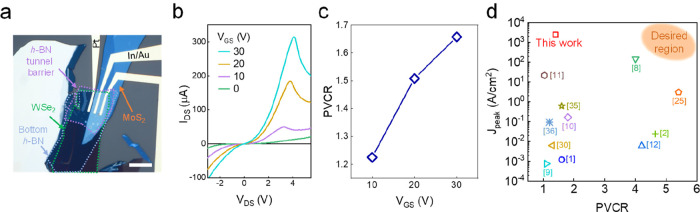
*V*_GS_-dependent NDR with *h*-BN tunnel barrier. (a) OM image of the heterostructure. The scale bar is 10 μm. (b) *V*_GS_-dependent NDR. (c) *V*_GS_-dependent PVCR and the (d) benchmark of *J*_peak_ versus PVCR for various reported 2D NDR devices based on tunnelling that operate at RT. The transverse tunnelling transistor^31^ is not included in this plot due to a different operation mechanism and lack of device size information.

The energy band alignment of the WSe_2_/*h*-BN/MoS_2_ device under varying applied *V*_DS_ is illustrated in Figure S8. When reverse bias (*V*_DS_ < 0) is applied, it allows the tunnelling of electrons from filled states in WSe_2_ through the *h*-BN barrier to reach the unoccupied states in the MoS_2_ CB. This tunnelling phenomenon results in a sharp increase in current as a larger negative *V*_DS_ is applied. Conversely, when positive voltage (*V*_DS_ > 0) is applied, the direction of carrier injection reverses. Electrons in the MoS_2_ CB tunnel through the barrier to reach the WSe_2_, leading to an inelastic tunnelling current. This current continues to increase until it reaches the maximum value, *I*_peak_. Beyond *V*_peak_, electrons from MoS_2_ face the midband gap state of the WSe_2_, where no density of states exists. Thus, the current decreases. Notably, some hot electrons may still overcome this barrier and contribute to the valley current (*I*_valley_). Subsequently, when thermionic emission provides sufficient energy for MoS_2_ electrons to transit to the WSe_2_ CB, as in a normal diode, the current once again begins to rise.

We extracted the PVCR, which exhibits an increasing trend as *V*_GS_ increases (Figure 4c). The overall PVCR value is not exceptionally high due to the presence of a substantial *I*_valley_. This can be attributed to a high probability of inelastic tunneling and thermally excited electrons across the tunnel barrier. Consequently, this results in a relatively small overall PVCR value. Nonetheless, the increase in *I*_peak_ surpasses the rise in *I*_valley_, leading to an elevation in PVCR as *V*_GS_ increases.

To benchmark our results, in Figure 4d we have plotted the *J*_peak_ (peak current density, *I*_peak_/device working area) and PVCR from other devices based on the heterostructure of 2D semiconductors.^1,2,8−12,25,30,35,36^ Among various types of devices employing different 2D materials, principles, and environments, we have selected devices based on tunneling that operate at RT. Our devices exhibit a large *J*_peak_ of 2420 A/cm^2^. A further increase in PVCR could be accomplished by exploring materials with different band gaps and higher doping levels, as well as carefully controlling the inelastic tunneling to minimize the *I*_valley_. It is noteworthy that the transverse tunnelling transistor demonstrated by Xiong et al.^31^ using black phosphorus exhibits a large *I*_peak_ and PVCR simultaneously. Employing transverse tunneling may be an alternative route to improve the PVCR.

In summary, our findings demonstrate a high *I*_peak_ NDR device by using vdW heterostructures. The utilization of In/Au and Pt vdW contacts facilitates the effective formation of the p–n junction. The devices show NDR, facilitated by F–N tunneling through a triangular barrier induced by applied *V*_GS_. Furthermore, integration of the *h-*BN tunnel barrier leads to improved NDR because of sharp band edges as well as inelastic tunnelling. This result yields high *I*_peak_ values. These results pave the way for potential applications in logic devices and oscillators.

## Supporting Information Available

Role of *h*-BN as a substrate, temperature-dependent transport of MoS_2_ and WSe_2_ with vdW contacts, OM image of the Cr/Au device, V_GS_-dependent *I*–*V* curves of the MoS_2_/WSe_2_ heterostructure with vdW contacts and Cr/Au contacts, *V*_DS_-dependent transfer curves of the MoS_2_/WSe_2_ heterostructure, temperature-dependence of the NDR device, thickness of each layer of the heterostructure, energy band diagram of the heterojunction, OM and *I*–*V* characteristics of additional NDR devices, experimental methods (PDF)
