# Supplementary material for: Room Temperature Negative Differential Resistance with High Peak Current in MoS2/WSe2 Heterostructures
Source: Nano Lett. 2024 Feb 16;24(8):2561–6. doi: 10.1021/acs.nanolett.3c04607 (PMC10906070; doi:10.1021/acs.nanolett.3c04607)
Supplement: Supplementary file 1 — nl3c04607_si_001.pdf [file nl3c04607_si_001.pdf]

Supporting Information

Room Temperature Negative Differential Resistance with High Peak Current in MoS_2/WSe_2 Heterostructures

*Jung Ho Kim[1], Soumya Sarkar[1], Yan Wang[1], Takashi Taniguchi[2], Kenji Watanabe[3], Manish Chhowalla[1,]**

[1]Department of Materials Science and Metallurgy, University of Cambridge, 27 Charles Babbage Road, Cambridge CB3 0FS, United Kingdom

[2]Research Center for Materials Nanoarchitectonics, National Institute for Materials Science, 1-1 Namiki, Tsukuba, Ibaraki 305-0044, Japan

[3]Research Center for Electronic and Optical Materials, National Institute for Materials Science, 1-1 Namiki, Tsukuba, Ibaraki 305-0044, Japan

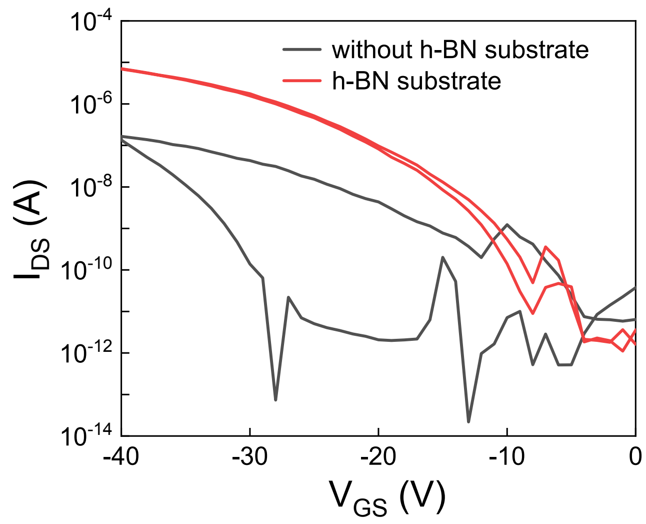

Figure S1. The role of *h*-BN as a substrate. The *h*-BN layer plays an important role in two aspects. First, it screens the electron doping effect that originates from the SiO_2 substrate, thereby enabling a high hole current. Secondly, it reduces the hysteresis by minimizing the effect of surface charge traps. Both devices were fabricated with Pt contacts.

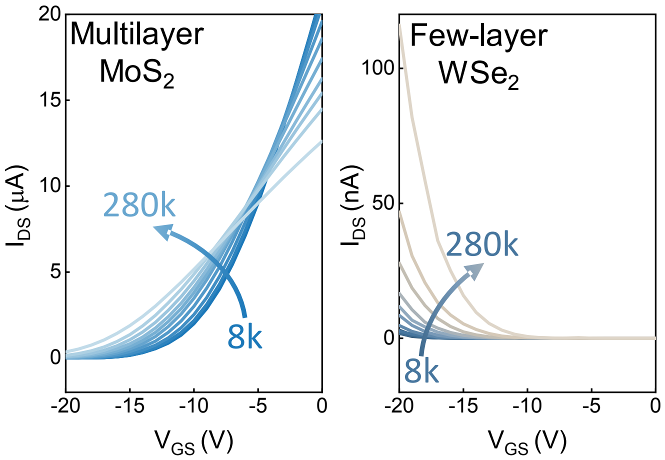

Figure S2. Temperature-dependent transport of MoS_2 and WSe_2 with vdW contacts. Linear scale transfer curves of MoS_2 with In/Au contacts and WSe_2 with Pt contacts under temperature sweep. The transfer curves show stable temperature dependence.

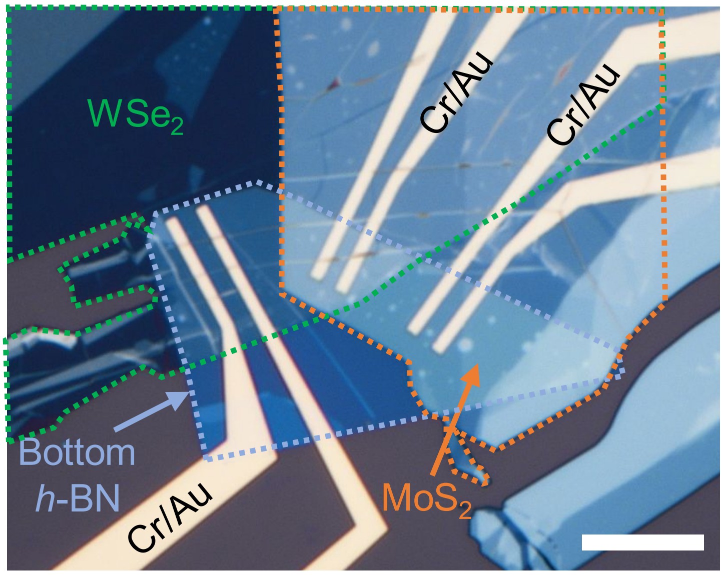

Figure S3. OM image of the Cr/Au device. MoS_2 and WSe_2 heterostructure on *h*-BN substrate, with Cr/Au contacts. The scale bar is 10 µm.

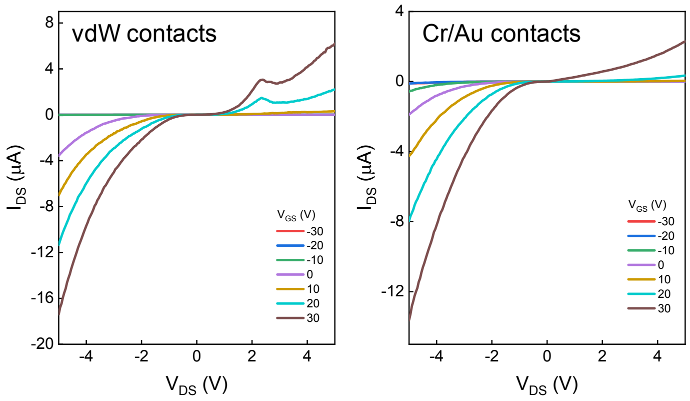

Figure S4. V_{GS}-dependent *I-V* curves of MoS_2/WSe_2 heterostructure with vdW contacts and Cr/Au contacts. The two graphs show a similar trend in the reverse bias region, but only In/Au contact enables the NDR effect.

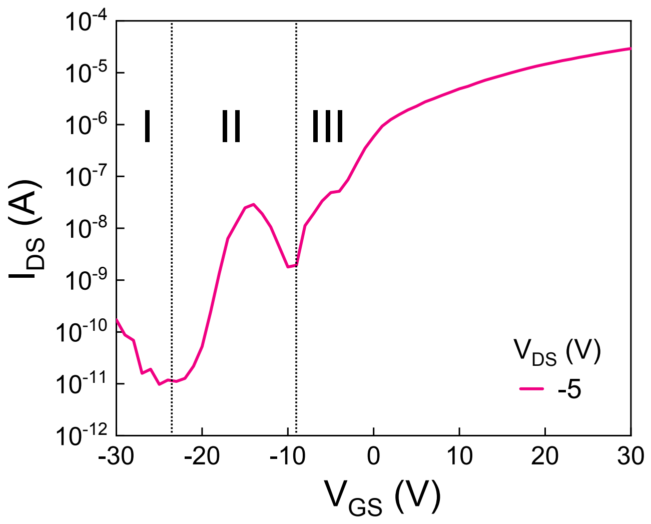

Figure S5. V_{DS}-dependent I_{DS}-V_{GS} curves of MoS_2/WSe_2 heterostructure. Below V_{GS}=-24 V (region I), the heterostructure device shows a low I_{DS} due to the depletion of MoS_2. This blocks the electron path because of highly resistive MoS_2. In region II (-24 V<V_{GS}<-9 V), both materials are in the subthreshold regime and reveal anti-ambipolar behavior, which peaks at V_{GS}=-14 V. This arises from the combined transport of decreasing hole conduction from WSe_2 and increasing electron conduction of MoS_2. Finally, in region III (-9 V<V_{GS}) the current increases with increasing electron conduction of both MoS_2 and WSe_2.

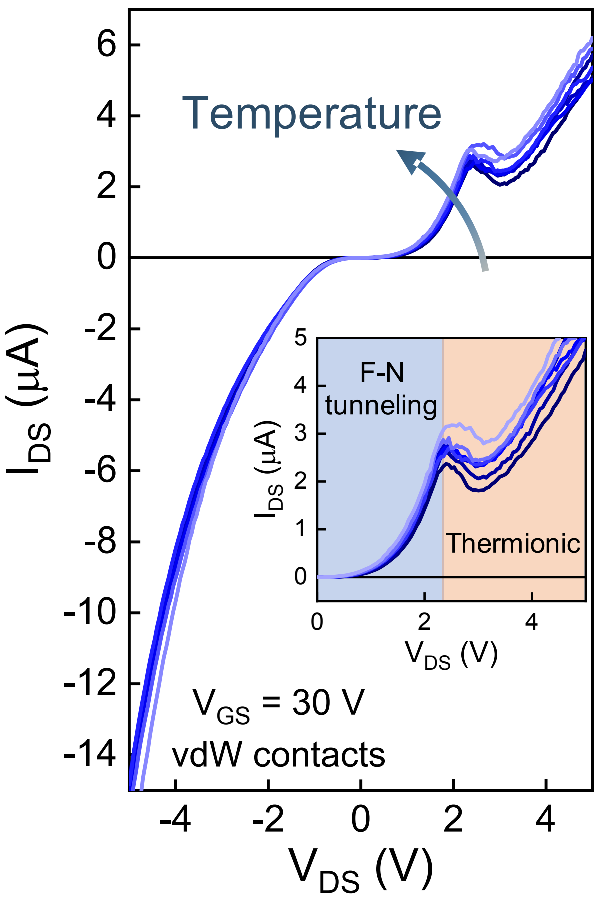

Figure S6. Temperature-dependence of NDR device. The temperature-dependent *I*-*V* curve of the NDR device. The temperature varies from 9 K to RT.

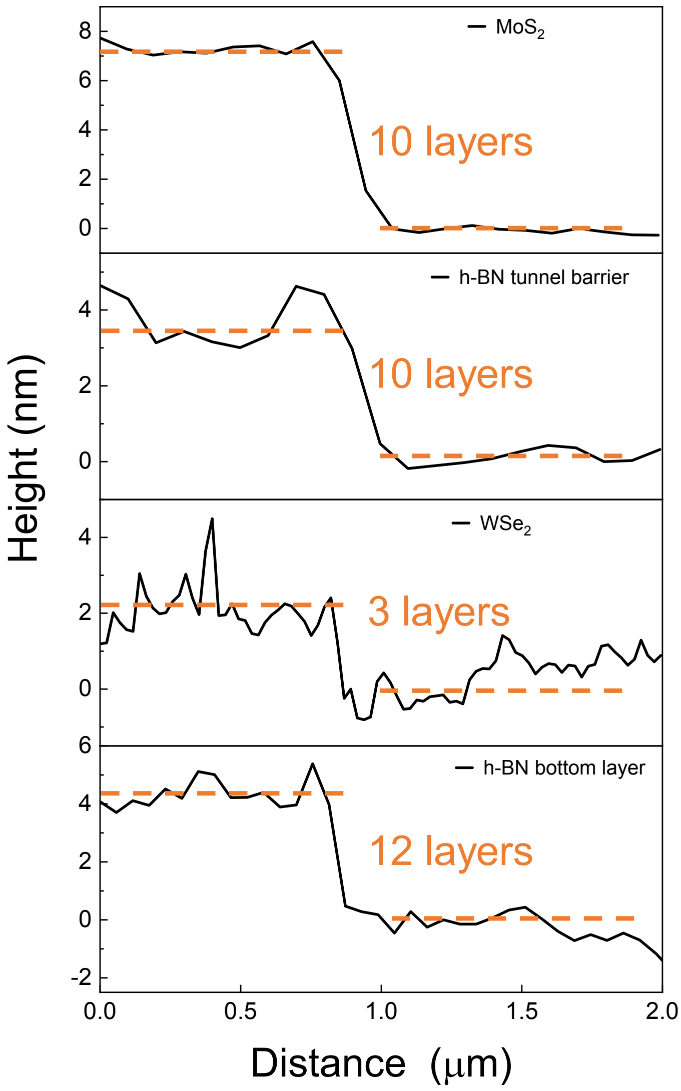

Figure S7. The thickness of each layer of the heterostructure. The thickness of each layer in the heterostructure was measured by AFM. The few-layer (3 layers) WSe_2 enables effective gate coupling, while multilayer (10 layers) MoS_2 is less affected by V_{GS}. Notably, the *h*-BN tunnel barrier is determined by a thickness measurement of 10 layers.

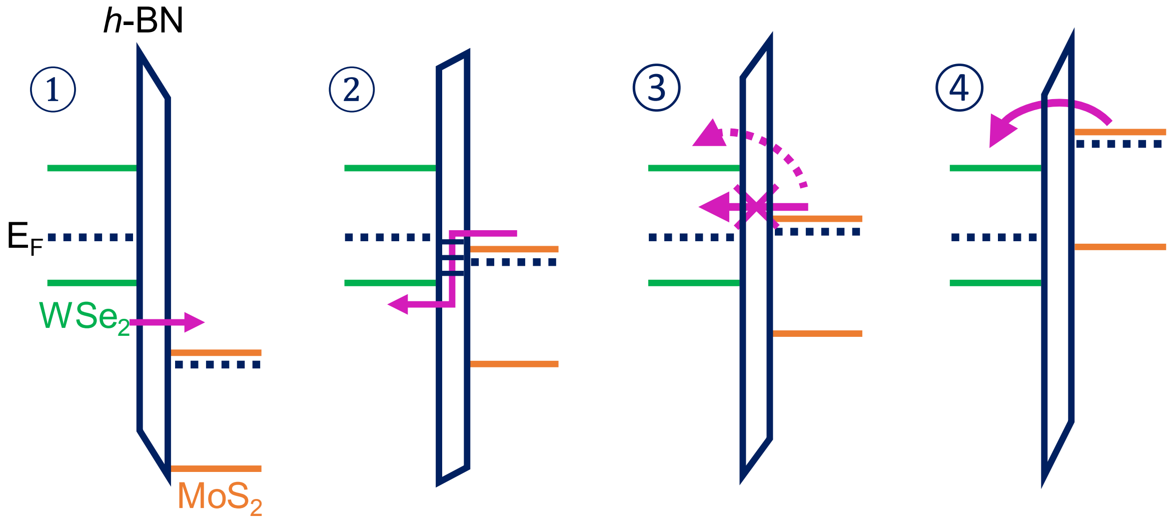

Figure S8. Energy band diagram of MoS_2/WSe_2 heterojunction with *h*-BN tunnel barrier. Step ① depicts the electrical transport at V_{DS}<0. The tunneling results in an increase of current. When 0<V_{DS}<V_{peak}, the energy band diagrams of the two materials start to overlap and form a type II junction. However, due to inelastic tunneling, an increase of current is observed (step ②). When additional V_{DS} is applied, the current decreases due to the excess overlap of the two energy bands, which is shown in step ③. However, some electrons with thermal energy can still overcome the barrier and reach the WSe_2 side. At step ④, thermally excited electrons contribute to the current.

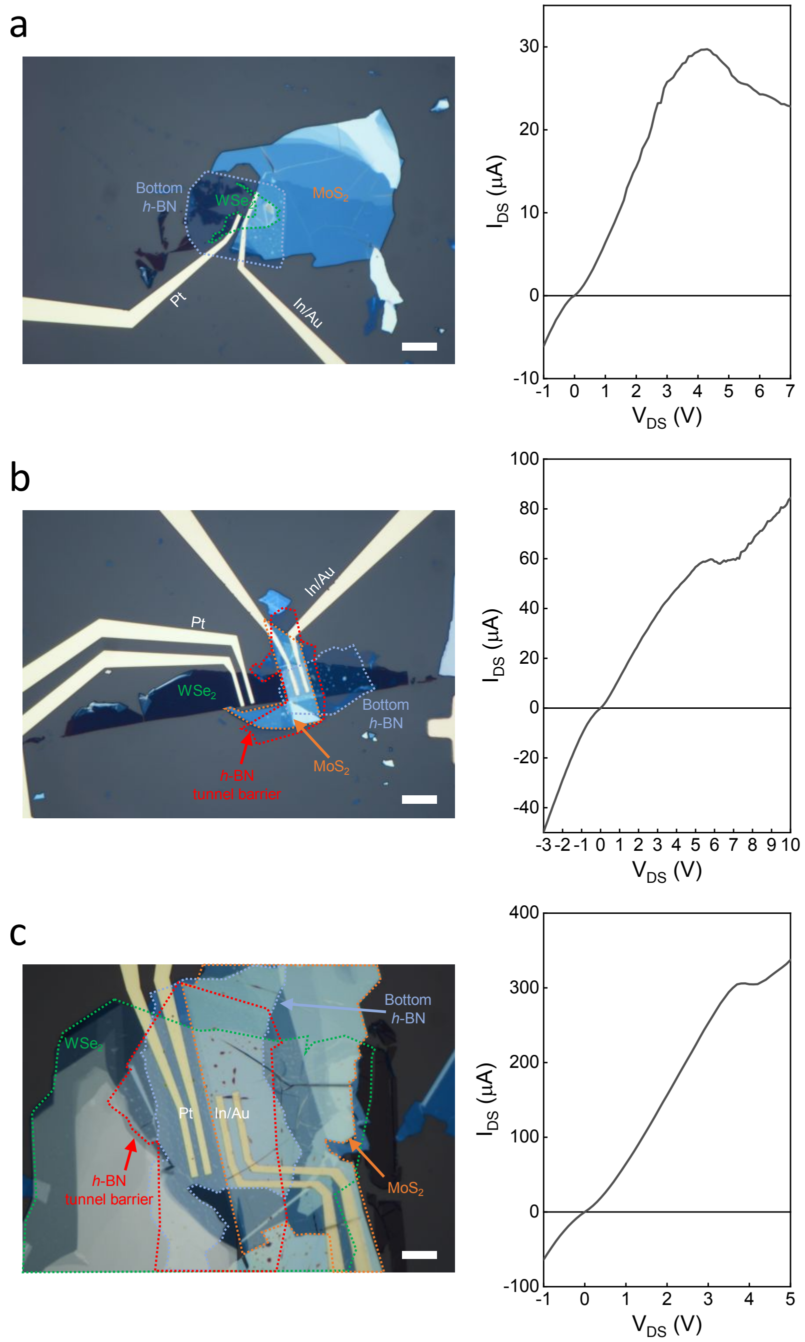

Figure S9. OM and *I-V* characteristics of MoS_2/WSe_2 NDR devices with vdW contacts. Additional NDR device demonstrations that are not presented in the main text. NDR behaviors of MoS_2/WSe_2 heterostructures (a) without and (b,c) with *h*-BN tunnel barrier. All scale bars are 10 μm.

Experimental Methods

Sample preparation and device fabrication

First, the *h*-BN bottom layer is mechanically exfoliated onto a pre-patterned 90 nm SiO_2 substrate with heavily doped Si for a global V_{GS}. MoS_2 and WSe_2 (HQ Graphene) flakes were obtained by mechanical exfoliation on a PDMS film (Gel-Pak). Once the thickness of the flakes was determined using OM, they were stacked on the as-exfoliated *h*-BN bottom layer, using a transfer stage at 40 ˚C. Subsequently, the samples were spin-coated with MMA/PMMA resist for electron beam lithography (EBL).

The first EBL step was carried out to pattern In/Au electrodes. In (8 nm) deposition was performed at a low rate of 0.1 Å, followed by a rapid deposition of Au at 1 Å.[14] To create an In/Au alloy, the samples were annealed in an Ar/H_2 environment at 200 ˚C. Following this, Pt electrodes were patterned using EBL, and Pt was deposited at a rate of 1 Å.[15] All metal deposition were performed at a base pressure of $<10^{-7}$ Torr.

Device measurement

Electrical transport measurements were conducted using a Keithley 4200 semiconductor analyzer under a high vacuum of $<10^{-6}$ Torr, using the Lakeshore cryogenic vacuum probe station. For low-temperature measurements, a temperature of 9 K was achieved by employing the closed-cycle compressor and Lakeshore temperature control system.
